# In-bore MRI-compatible transrectal ultrasound and photoacoustic imaging

**DOI:** 10.1016/j.pacs.2025.100779

**Published:** 2025-11-01

**Authors:** Ryo Murakami, Yang Wang, Wojciech G. Lesniak, Ryosuke Tsumura, Yichuan Tang, Shang Gao, Yasuyuki Tsunoi, Christopher J. Nycz, Martin G. Pomper, Gregory S. Fischer, Haichong K. Zhang

**Affiliations:** a Robotics Engineering, Worcester Polytechnic Institute, MA, United States; b Biomedical Engineering, Worcester Polytechnic Institute, MA, United States; c Computer Science, Worcester Polytechnic Institute, MA, United States; d Department of Radiology, University of Texas Southwestern Medical Center, TX, United States; e Division of Bioinformation and Therapeutic Systems, National Defense Medical College Research Institute, Saitama, Japan

**Keywords:** MRI-compatible, Prostate cancer, Transrectal, MRI-compatible robotics, Tri-modal imaging, Three-dimensional imaging

## Abstract

Prostate cancer (PCa) remains one of the leading causes of cancer-related mortality in males. While MRI is widely used for PCa diagnosis due to its high sensitivity, it is limited by its poor specificity in detecting aggressive PCa. Molecular targeted photoacoustic (PA) imaging is a non-ionizing technique known for its potential to achieve both high sensitivity and specificity. It also provides real-time imaging capability, which complements MRI’s limitation of slow imaging speed during intraoperative image-guided procedures. This research presents a tri-modal imaging system that integrates MRI, PA, and ultrasound (US) to enhance PCa diagnosis and image-guided procedures. We introduce an MRI-compatible PA/US imaging platform featuring a reflector-based transrectal probe with an integrated optical fiber delivery channel. The probe’s MRI-compatible actuation system enables 3D PA/US imaging in parallel with MRI scanning. Comprehensive performance evaluation included phantom studies to assess imaging quality, MRI compatibility, and *in vivo* validation. Results demonstrated successful tri-modal imaging capabilities with acceptable MRI artifacts and confirmed the system’s effectiveness for spectroscopic PA imaging with an exogenous contrast agent. The platform functions during active MRI scan sequences, enabling rapid target visualization without requiring patient repositioning between MRI and PA/US suites. These findings support the feasibility of in-bore MRI-compatible PA/US imaging and demonstrate its potential for clinical translation in the diagnosis and management of PCa.

## 1 Introduction

Prostate cancer (PCa) continues to be one of the most frequently diagnosed cancer types and ranks as the second leading cause of cancer-related deaths among males [1]. Timely detection of PCa significantly increases the effectiveness of treatment and improves patient outcomes. The established diagnostic process for PCa involves screening methods such as prostate-specific antigen (PSA) tests [2], [3], needle biopsy for definitive diagnosis, and staging evaluation using the Gleason score [4]. Imaging techniques including ultrasound (US) and magnetic resonance imaging (MRI) are employed for initial staging, therapy follow-up, and detecting recurrence. Although positron emission tomography/computed tomography (PET/CT) with receptor-targeted radiotracers is a recently introduced alternative for imaging PCa [5], its ionizing nature presents an inevitable limitation. Despite the US offering real-time visualization, it is not tumor or lesion-specific, leading to a systematic biopsy approach, instead of targeted [6]. While MRI is known for its wide field of view [7], it suffers from its limited specificity, additionally, it is not capable of providing a sufficient frame rate for real-time intraoperative biopsy guidance; therefore, imaging is usually performed prior to the biopsy procedure to guide it with the static preoperative image [8]. Similar to MRI, PET/CT also suffers from its limited frame rate and is not suitable for intraoperative guidance [7]. Given the inherent limitations of current imaging techniques, there is a need to develop an imaging modality that offers non-ionizing, real-time PCa imaging with high-sensitivity and specificity.

Molecular targeted photoacoustic (PA) imaging is an emerging non-ionizing imaging technique that integrates the benefits of optical and US imaging [9], [10]. Spectroscopic PA (sPA) provides quantification of multiple indices, offering valuable insights into cancer severity and prognosis [11], as well as therapeutic monitoring [12], [13], [14], [15]. Prostate-specific membrane antigen (PSMA) is a receptor on the surface of PCa cells [16], [17]. This receptor is characterized by its strong correlation with aggressive tumors [18], [19]. In addition to its functional imaging abilities, PA imaging has demonstrated its capability in real-time monitoring [20]. These attributes position PA imaging as a promising modality for PCa diagnosis. In fact, there is research combining PA and US for PCa imaging [21], [22] to add enhanced specificity to US imaging.

Given the advantages of PA, the integration of PA imaging with MRI can be a promising solution to address MRI’s limitations in specificity and frame rate of MRI while maintaining its non-ionizing properties.

**Fig. 1:**
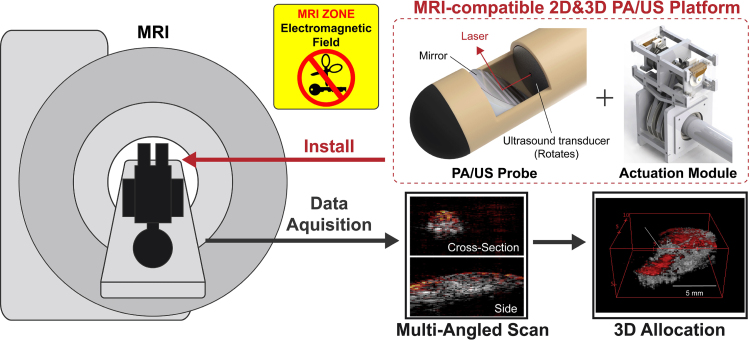
Proposed MRI-compatible PA/US imaging platform and its workflow. The PA/US device is made to be MRI compatible to enable 2D and 3D imaging inside an MRI scanner, which avoids patient relocation and enables repeated MRI scans even during PA/US imaging. The MRI-compatible actuation module allows precise angular control inside the MRI scanner. MRI and PA/US are expected to complement their limitations each other.

Here, we propose to develop an MRI-compatible PA/US imaging platform. The overall concept is illustrated in Fig. 1. This PA/US imaging system is placed inside the MRI bore, enabling these three imaging modalities to be performed without patient repositioning. With the introduction of MRI-compatible PA imaging into clinical settings, a potential workflow is to overlay PA/US with MRI. In this workflow, MRI serves as the primary imaging modality, with PA/US images used as needed to supplement the MRI with additional information. Although the procedure is guided primarily by MRI, PA/US images can visualize locally important structures such as nerves and blood vessels, and more importantly, can detect PCa with higher specificity than MRI. Another potential workflow is using PA/US as the primary imaging modality and using MRI as a global map. This approach enables on-demand updates of MRI images in parallel with local real-time PA/US imaging, which was unattainable when the PA/US device lacked MRI compatibility. We anticipate that by leveraging the supplementary role of MRI, this system can be adapted to accommodate large subjects, thereby facilitating further exploration of the clinical translation.

Ensuring MRI compatibility is integral for the realization of our proposed system, as highlighted in the concept. Achieving this compatibility presents several challenges due to the inherent attributes of the MRI scanner, including the magnetic field, time-varying magnetic field gradients, and radio frequency pulses, as well as the MRI’s sensitivity to external interference [23]. These challenges include the strict prohibition of magnetic materials and the requirement to minimize non-magnetic metallic components to avoid potential risks like induced thermal effects and artifacts in MRI images. The domain of MRI-compatible devices, such as robotics, has witnessed considerable research efforts, with several groups focusing on addressing these limitations [24], [25], [26], [27], [28]. Researchers have made several efforts to make the US transducer MRI-compatible for imaging and therapeutic purposes [7], [29], [30]. For MRI-compatible PA imaging, Chen, Gezginer et al. developed an MRI-compatible PA tomography system for a pre-clinical MRI scanner [31], [32]. Although these studies focus on MRI-compatible PA imaging, they are not designed for clinical applications such as transrectal imaging configuration.

This paper introduces the design of an MRI-compatible transrectal PA/US imaging platform. This platform integrates a custom-made MRI-compatible PA/US probe with its associated actuation module. The performance of the newly developed system is evaluated using both phantom models and an *in vivo* setup. The contributions of this study can be summarized as follows:

• Design and development of a transrectal MRI-compatible PA/US platform including its actuation module. To the best of our knowledge, this is the first transrectal MRI-compatible PA/US imaging platform to be introduced.
• Investigation of the feasibility of performing in-bore tri-modal imaging using the developed platform, phantoms, and *in vivo* subjects.

The remainder of this manuscript is structured as follows: Section 2 primarily describes the design of the MRI-compatible PA/US probe, its actuation module, the experimental setup, and the evaluation metrics. The results are compiled and explained in Section 3. Section 4 discusses the performance of the developed system, potential applications, and limitations based on obtained results. Lastly, Section 5 concludes the paper.

## 2 Materials and methods

### 2.1 Hardware basics

#### 2.1.1 Design policy for MRI compatibility

The electromagnetic fields generated by MRI are extremely powerful, and officially, there are three categories defined for devices used within MRI environments: MR safe, MR conditional, and MR unsafe [33]. The PA/US probe and its actuation module in this study were designed to be MR conditional. MR conditional refers to a category in which a device can be safely used within the MRI environment under specific conditions. In actual medical settings, a number of devices categorized as MR conditional are routinely used within MRI systems [34]; therefore, being categorized in MR conditional would not hinder the actual clinical use of the proposed devices. To achieve this, all the components of the devices are required to be non-magnetic and non-ferromagnetic. The entire device including its cabling only consists of well-known MR-compatible materials including brass, chrome, and aluminum.

#### 2.1.2 Customized MRI-compatible PA/US probe

**Fig. 2:**
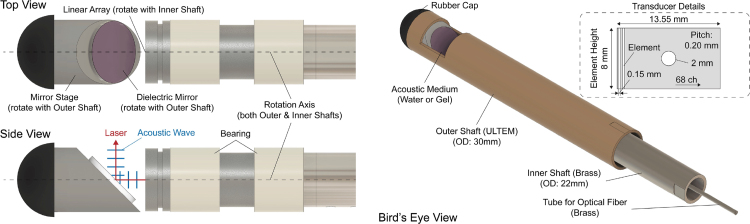
Design of MRI-compatible PA/US probe. (Left) Top view and side view of the probe without the outer shaft, (Right) Bird’s-eye view of the probe with the outer shaft. (The design of the outer shaft and the use of the rubber cap may vary among the experiments.), (Top Right) Transducer details.

A customized MRI-compatible PA probe is developed (Fig. 2). It consists of two parts: the outer shaft and the inner shaft. The outer shaft is made of ULTEM1010 (ULTEM 1010, Natural, Solid Infill, Fused Deposition Modeling (FDM), Xeometry, Maryland, United States), and the inner shaft is made of brass. Between the two shafts, bearings made of glass-filled PTFE (${\text{iglide}}^{\text{®}}$ i3-PL, igus, Inc., Rhode Island, United States) are placed to smoothen the rotation. On the distal side of the inner shaft, a 68-element linear array transducer (68-element linear array transducer (element pitch: 0.2 mm), Japan Probe Co, Ltd., Kanagawa, Japan) is fixed and rotates with the inner shaft. The transducer has a hole (diameter: 2 mm) at the center of the array so that an optical fiber (FT1000EMT, Thorlabs, New Jersey, United States) can pass through it and shoot a laser. A dielectric mirror (25 mm Diameter 750–1100 nm Broadband λ/10 Mirror, Edmund Optics Inc., New Jersey, United States) stand is fixed to the outer shaft and the mirror has a 45-degree slope with respect to the horizontal axis. The mirror is designed so that it can reflect both the acoustic wave and the laser, altering their trajectories by 90 degrees. The cable that connects between the probe and its signal acquisition system is a well-shielded cable, with the shield connecting the ground of an isolated power supply. The specifications of the transducer and the field parameters used in experiments are summarized in Table 1. As an acoustic medium, either water or ultrasound gel fills the space between the transducer and the mirror, as specified in each experiment.

**Table 1:** Transducer and field parameters used in both experiment and simulation.

Parameter	Notation	Value	Unit
Speed of sound	c	1490	m/s
Central frequency	$f_{0}$	10	MHz
Sampling frequency (US)	$f_{su}$	40.0	MHz
Sampling frequency (PA)	$f_{sp}$	62.5	MHz
Number of elements	N	68	–
Element pitch	p	0.2	mm
Element width	w	0.15	mm
Element height	h	8	mm
Curvature for elevational focus	R	45	mm

#### 2.1.3 Probe actuation system

**Fig. 3:**
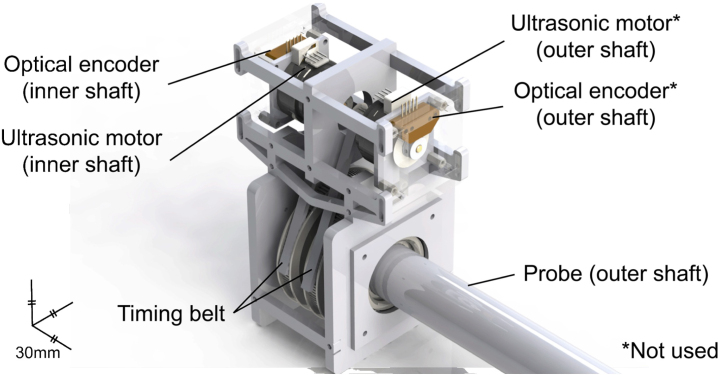
CAD model of the actuation module with PA probe mounted.

An MRI-compatible probe actuation system is developed and fabricated with MRI-safe material and MRI-conditional electronics. Fig. 3 represents the CAD model of the developed actuation module. A dedicated control box and communication flow are also designed for the module.

An ultrasonic motor (USR30-S4N, SHINSEI Corporation, Tokyo, Japan) is used as an actuator, ensuring its compatibility with MRI by precluding the use of magnetic materials. A timing belt system is used to transfer the movement from the motor to the inner shaft, with a predetermined ratio of 5:1. A 1250 CPR optical encoder (E2-1250- 157-IE-H-D-1, US Digital Corporation, WA, United States) is mounted on the back shaft of the motor to monitor the rotation of the shaft, which is converted corresponding to the predetermined ratio.

### 2.2 Rotational 3D scanning and image formation

**Fig. 4:**
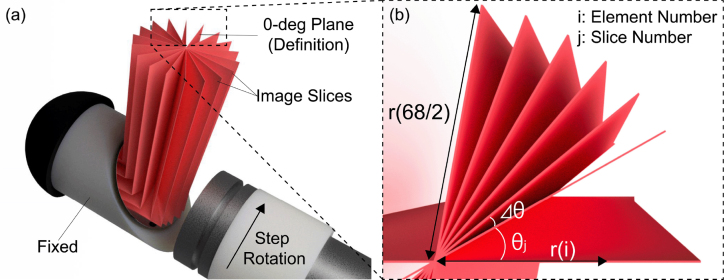
Schematics of the targeted 3D image scanning & reconstruction. (a) Bird’s-eye view, (b) Top view of the image slices.

The developed imaging probe is designed so that the rotation of the linear transducer enables the rotational 3D scan (Fig. 4(a)). The acquired imaging slices are allocated to their corresponding angles (Fig. 4(b)). Here, i is the transducer element number from the center of rotation and j is the slice number. Since the linear array has 68 elements in total, the maximum number of i is 34. The 3D data is generated in the MATLAB environment (MATLAB, The MathWorks, Inc., Massachusetts, United States), and the elevational synthetic aperture focusing (SAF) is applied to both 3D US and PA data [35]. During the rotation, the mirror attached to the outer probe shaft is fixed. For 3D imaging in this specific study, the laser wavelength of 800 nm is used as a representative wavelength to cause the PA effect on both oxy and deoxyhemoglobin.

### 2.3 Spectral decomposition

For the purpose of functional PA imaging, a technique called spectral decomposition is utilized in this study. The received PA signals can be assumed as linear combinations of multiple absorbers in a tissue or contrast agents; therefore, the obtained signals can be decoupled into the contribution of each absorber by referring to their absorbing characteristics. This concept can be formularized as shown in Eq. (1).

(1) $${\text{argmin}}_{m_{1,2,\ldots ,M}}\|\underset{w}{\sum }\left(p-\overset{M}{\underset{i=1}{\sum }}m_{i}\mu_{a,i}\right)\|$$

where p is the obtained PA spectrum, $\mu_{a,i}$ and M are the absorption spectrum of the contrast i and M is the number of assumed optical absorbers. w is the applied laser wavelength. $m_{i}$ as the weight of the contract i, is calculated as an output of this equation [19].

### 2.4 Experimental setup

#### 2.4.1 System architecture

**Fig. 5:**
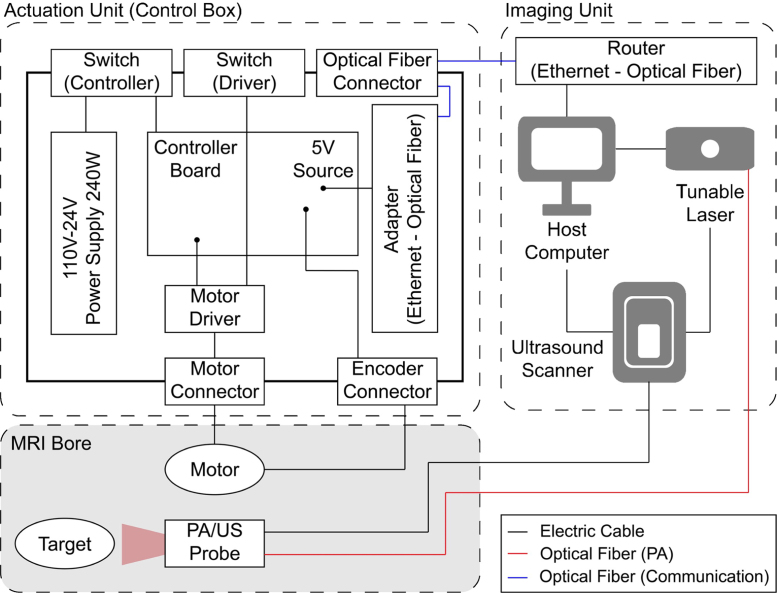
System Architecture involving Actuation Unit and Imaging Unit.

Fig. 5 shows the system architecture, especially for the in-bore setting. The PA/US probe, a target sample, and the actuation module consisting of the ultrasonic motors and the encoders are placed in the MRI bore (SIGNA Premier 3.0T MRI scanner, GE Healthcare Technologies Inc., Chicago, IL, United States), and the control box includes a power source, drivers, and a controller board. As the laser device (Phocus MOBILE, OPOTEK, Inc., Carlsbad, CA, United States) is used and the ultrasound data acquisition system (Vantage 128, Verasonics, Kirkland, WA, United States) is located in the closet room, which is next to the MRI room and shielded from the MRI-induced electromagnetic field. The cables required for the actuation module and transducer, along with the optical fiber necessary for the laser system, are passed through the brass partition that separates the equipment room from the MRI room.

A control box is comprised of the controller (DMC-4143-CARD, Galil Motion Control, Inc., Rocklin, CA, United States) for the actuation module, the drivers (D6030/24V, SHINSEI Corporation, Tokyo, Japan) for the motors, the media converter that transforms optical signal to serial signal, and two power supplies. The communication between the control box and the computer is established using a group of optical fiber cables, a media converter, and a router that acts as the hub of the communication network. The control box was positioned in the equipment room, rather than the MRI room for safety in this particular experiment; however, the optical communication system is designed with the intent of allowing the control box to be located in the MRI room, thereby potentially facilitating additional noise reduction.

#### 2.4.2 Sample preparation

**Fig. 6:**
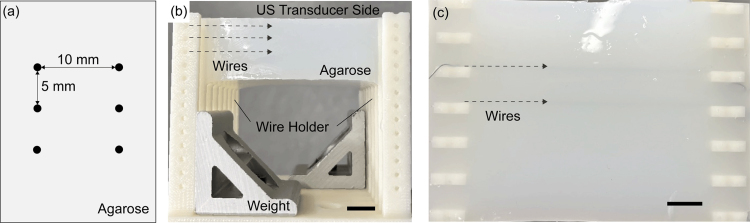
Design and photograph of the wire phantom with optical scatters; (a) Cross-section of the designed phantom, (b) Side view, and (c) Top view of the fabricated phantom (Scale bar: 10 mm).
• Tube Phantom with Contrast Agents
  Three tubing targets are prepared to evaluate the MRI compatibility of the developed system and its tri-modal imaging capability. It employs indocyanine green (ICG) solution (Indocyanine green 250 mg, U.S. Pharmacopeia, United States) and radiation water (${\text{MR-SPOT}}^{\text{®}}$ 121, ${\text{Beekley Medical}}^{\text{®}}$, Connecticut, United States). Each silicone tube (Outer diameter (OD): 2 mm, Inner diameter (ID): 1 mm) encapsulates either 25μM ICG solution or ${\text{MR-SPOT}}^{\text{®}}$ exclusively, or a 2:1 mixture of 25μM ICG and ${\text{MR-SPOT}}^{\text{®}}$. The configuration of the tubes is depicted in Fig. 11(a) and their cross-sections are scanned with MRI, US, and PA employing ultrasound gel as the coupling medium. With this setup, it can be hypothesized that solely PA imaging can distinguish the tubes containing ICG based on the ICG-specific optical absorbance characteristics. The wavelengths shot for this phantom range from 700 to 850 nm with the 10 nm steps.

• Wire Phantom with Optical Scatters (3 × 2 grid)
  For the analysis of a signal-to-noise ratio (SNR) and resolution, the wire phantom with optical scatters is designed as shown in Fig. 6 (a). The wire phantom consists of six parallel-aligned black wires (Diameter: 0.2 mm), which serve as point targets. In the horizontal and vertical directions, the distance between adjacent wires is 5 mm and 10 mm, respectively. The wires are fixed in an agarose-based medium, which is a mixture of 300 g of water, 4 g of whole milk, and 12 g of agarose powder (AGAROSE Molecular Biology Grade (IB70041), IBI Scientific, Iowa, United States).

**Fig. 11:**
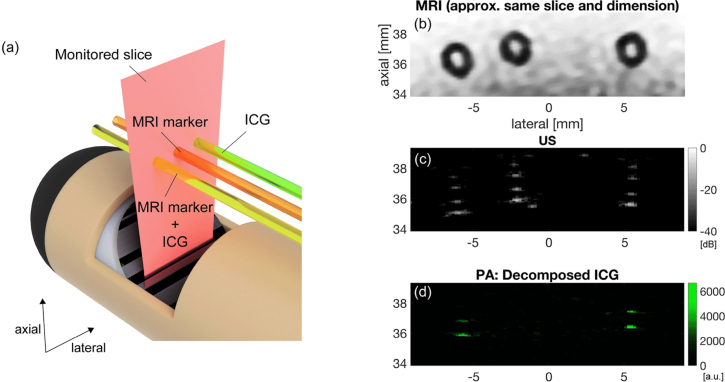
(left, a) Configuration of the tube phantom consisting of 25 μM ICG, MRI marker, Mixture of 25 μM ICG ＋ MRI marker (mixing ratio: 2:1), (right) Result of the 2D PA/US imaging and the MRI scan. (b) MRI image, (c) US, (d) Decomposed ICG in sPA. (The registration of the MRI image to PA/US was manually performed based on the geometrical relationship between the probe geometry captured by MRI and the PA/US imaging window. The alignment of (b) was done manually based on the US image. The registration and alignment process has a certain error.).
• *in vivo* Mice Subjects
  Living mice are employed to evaluate the feasibility of the *in vivo* imaging capability of the developed system. The mice are first anesthetized using isoflurane and then secured onto the imaging stage. During the experiment, anesthesia is continuously supplied via a nose cone, and the animals’ respiration and body temperature are regularly monitored. The stage is in contact with warm water maintained at approximately 37 °C to ensure the mice’s thermal regulation. Water is used as the acoustic medium between the probe and the stage, and ultrasound gel is applied between the stage and the mice. Cyanine7.5 (Cyanine7.5 NHS ester (Cat.# 46020, Lumiprobe Corporation, Maryland, United States)) is used as a PA contrast agent when needed and is administered to mice via tail vein injection at a concentration of 510 μM. For spectroscopic PA imaging, the laser wavelengths of 700 to 850 nm are used with the 10 nm step. 3D PA imaging employed 800 nm laser wavelength to obtain signals from both oxy and deoxyhemoglobin. This *in vivo* study was approved by the Institutional Animal Care and Use Committee (IACUC) at Worcester Polytechnic Institute [Protocol number: 23-155].

### 2.5 Evaluation metrics

#### 2.5.1 Indices for image quality evaluation

To quantify the quality of the acquired 2D images, an SNR and a resolution are computed in this study by visualizing the grid wire phantom. Resolution is computed as the full width at half maximum (FWHM). SNR is defined as shown in the following Eq. (2).

(2) $$\text{SNR}=20\text{log}\frac{\left|I_{\text{max}}\right|}{\sigma_{\text{noise}}}$$

where $I_{\text{max}}$ is the peak signal intensity at a target and $\sigma_{\text{noise}}$ represents the standard deviation of signal intensities in an experimentally defined noise area [36].

#### 2.5.2 Evaluation criteria for MRI compatibility

In accordance with the criteria for MRI compatibility as delineated by Tsekos *et al*. [23], the criteria for a device to be deemed MRI compatible within the context of this research are defined as follows with the designed experiment for each evaluation. One of the examples of the parameters used for the MRI scan in this experiment is summarized in Table 2.

**Table 2:** MRI acquisition parameters used in the *in vivo* study.

Parameter	Value
Sequence	MP-RAGE
TR (ms)	2562.35
TE (ms)	2.932
TI (ms)	940
Slice thickness (mm)	0.79
Center frequency (kHz)	127.7476
Pixel bandwidth (Hz)	122.07
Spacing (mm)	0.4

**Criterion 1:** **The device can be safely placed in the MRI environment. (e.g., magnetic force)**
  - The probe and the actuation module are placed inside the MRI bore and confirm if any force is induced.
**Criterion 2:** **The presence of the device does not significantly affect the MRI image quality.**
  - The MRI imaging is conducted around the image slice of PA/US with the probe and the actuation module placed inside the MRI bore.
**Criterion 3:** **The operation of the device is not affected by the MRI scanner.**
  - PA/US imaging and actuation are performed inside the MRI and their performance is evaluated.

## 3 Results

### 3.1 Resolution & SNR evaluation of PA/US

We evaluated the image quality of US and PA imaging using the wire phantom with optical scatters (Figs. 8(b) and (c)) as the imaging performance of the developed system. Optical scatters were added to evaluate the optical attenuation of the developed illumination mechanism. In both imaging methods, six-point targets as the cross-section of wires were visualized. In order to investigate the angular dependency of the image quality, the inner shaft with the US transducer was controlled to have both 0 deg and 90 deg rotation. Here, the case of 0 deg is illustrated in Fig. 8(a). The set of wires is rotated with the transducer to be visualized as point targets in US/PA images. For each angle, a total of four scans were performed for both US and PA imaging. The laser wavelength was 720 nm and a 1000-frame averaging filter was applied.

SNR and FWHM were computed for statistical analysis and summarized in Fig. 9. For US imaging at 0 degrees, SNR values of 40–50 dB were constantly observed across all positions, with stable FWHM values of approximately 0.2 mm. At 90 degrees, a depth-dependent degradation was noticed both in SNR and FWHM. On the other hand, PA imaging demonstrated relatively lower SNR values of 30–35 dB across both angles, with slightly higher values at the top position. It is noteworthy that, although the optical scatterers were used in the agarose phantom, the SNR values in PA imaging were not significantly affected by the depth within the 10 mm region.

### 3.2 MRI compatibility evaluation

In order to confirm the MRI compatibility of the developed system, including the imaging probe and the actuation module, the evaluation was performed along with the three criteria as defined in Section 2.5.2.

#### Criterion 1: Safe installation

We confirmed the safe installation of the developed system, including the PA/US probe and the actuation module, into the MRI bore, and no significant movements of the system attributable to the MRI scanner observed throughout the experiment. Consequently, we can affirm that the first criterion for MRI compatibility has been successfully fulfilled.

#### Criterion 2: MRI image quality

To evaluate how much the developed system affects the quality of MRI images, the MRI image obtained in the tube phantom experiment was analyzed, specifically the amount of artifacts in the MRI image (Fig. 11(a)). Although artifacts caused by the metals in the developed system were observed, these were sufficiently minimal to enable visualization of the tube phantoms which is located around 10 mm away from the outer shaft wall of the probe. This result suggests that Criterion 2 is met, given that a target maintains a specific distance from the probe’s outer shaft wall to avoid artifact interference.

#### Criterion 3: System performance

For this criterion, both the performance of the actuation module and the imaging unit need to be evaluated. The actuation module was controlled inside the MRI bore to assess its performance in the MRI environment. As a control target, 1 deg/step, up to 182 degrees was applied to the inner shaft. The result shown in Fig. 10(c) indicates that the rotation control functioned as expected, confirming the MRI compatibility of the developed actuation module. Note that the actuation was performed under the MRI scanning sequence, implying the capability of simultaneous MRI scanning and probe actuation.

The SNR values were measured to evaluate the MRI compatibility of PA and US imaging in cases where the MRI sequence was both on and off (Fig. 10(a–b)). The tail of *in vivo* mouse was used as the imaging target. The SNR values were computed by taking the mean along the y direction and treating it as one-dimensional data. The results indicate that practical contrast values were achieved when the MRI was both on and off. Furthermore, the similarity between the on and off conditions suggests that same as actuation, PA/US imaging can also be feasible to perform simultaneously with MRI scanning.

Based on the evaluation above, it can be claimed that Criterion 3 is satisfied for both actuation and PA/US imaging.

Since the three criteria for MRI compatibility have been satisfied as discussed, it can be concluded that the MRI compatibility of the developed system with the configuration is confirmed based on the definition.

### 3.3 Phantom evaluation of in-bore tri-modal imaging

For the evaluation of the tri-modal imaging capability of the entire system, PA, US, and MRI imaging were performed (Fig. 11(b–d)). The PA/US probe and the actuation module were placed inside the MRI bore throughout the tri-modal imaging. Once the MRI slice is found, the image is manually scaled to be aligned with the US image. Although both MRI and US can depict all the tube cross-sections, the circles with holes are most clearly revealed in the MRI image. The US image captures the inner and outer surface of tubings via acoustic reflection, while the PA image depicts signals from the contrast agent appearing at the inner surface. Taking into account the geometry of the tube (OD: 2 mm, ID: 1 mm), it is confirmed that the registered scale of the MRI image is appropriate by comparing it with the scales of PA and US.

As demonstrated in Fig. 11, MRI, US, and PA imaging were achieved without any change in the pose of the imaging target and the imaging device. This eliminates potential organ deformation and alignment complications, realizing more reliable image registration.

Spectroscopic decomposition with respect to the ICG spectrum was carried out to assess the performance of sPA imaging, as shown in Fig. 11(d). The sPA result distinctly emphasizes the two tubes holding the ICG solution. This result demonstrates the device’s ability for selective visualization of PCa, which is the expected function of PA imaging for enhanced specificity of the tri-modal imaging.

### 3.4 *in vivo* imaging capability evaluation

**Fig. 7:**
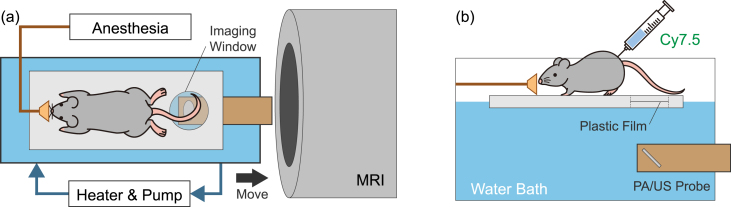
Illustration showing the *in vivo* experiment setup; (a) Top view, (b) Side view.

In order to evaluate the *in vivo* imaging capability of the developed system, the two types of *in vivo* study were designed and conducted: (1) *in vivo* spectroscopic PA & US imaging, and (2) In-bore tri-modal 3D imaging. The result of each study is summarized in the following subsections. In both studies, the tail of the mice was imaged as the target (Fig. 7). This is because it has a relatively simple anatomy, such as the abdomen, which allows reliable imaging alignment.

**Fig. 8:**
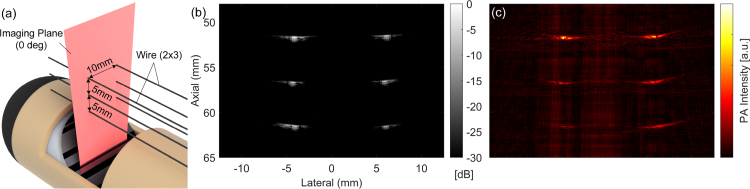
(a) Experimental setup of the wire phantom with optical scatters (0 deg), (b) US image (0 deg), (c) Single-wavelength (720nm) PA image (0 deg).

**Fig. 9:**
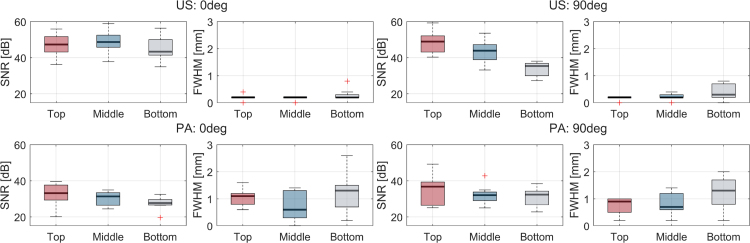
SNR and FWHM in the optical scattering medium, (Top: US, Bottom: PA, Left: 0 deg, Right: 90 deg).

**Fig. 10:**
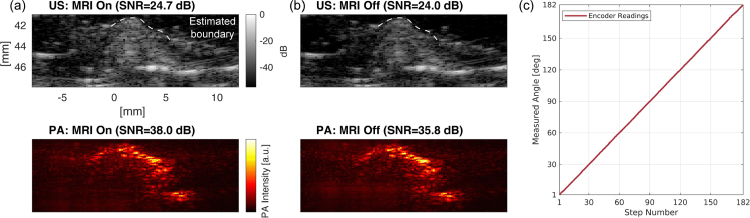
MRI compatibility evaluation for Criterion 3; (a–b) Comparison of in-bore PA/US imaging quality between the cases of MRI sequence is on and off. (PA single wavelength: 700 nm, imaging target: cross-section of mouse tail), (c) Performance of actuation module inside MRI.

#### 3.4.1 Spectroscopic PA & US imaging

**Fig. 12:**
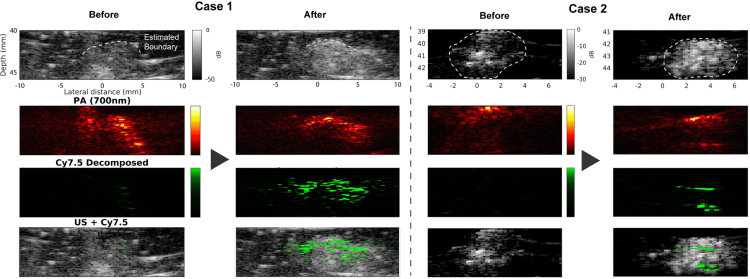
Results of *in vivo* spectroscopic PA imaging for two cases (imaging target: cross-section of mouse tail).

This specific study aims to evaluate the spectroscopic PA and US imaging capability *in vivo*. The mouse was prepared as described in Section 2.4.2. Once the mouse was ready, US and spectroscopic PA imaging were performed as pre-injection imaging. Then, the prepared Cy7.5 agent was injected through the tail vein followed by spectroscopic PA imaging as post-injection imaging. Both PA data were processed by the spectral decomposition technique to extract the Cy7.5-oriented spectrum. Other components considered for the decomposition were oxyhemoglobin, deoxyhemoglobin, and myoglobin. For quantitative comparison, the decomposed value to Cy7.5 was scaled based on the sum of the blood-oriented spectra. The results of this experiment are shown in Figs. 12 for two different cases.

In both before and after injection, the cross-section of the tail is visualized in the US and single wavelength PA at 700 nm, while not exactly the same cross-section was imaged due to the contrast agent injection between the two scans. The spectral decomposition technique gave the result that the decomposed value of Cy7.5 in the case of after injection is much higher than the one before injection, which is consistent across the two cases, supporting the spectroscopic imaging capability of the developed system, including *in vivo* environment.

#### 3.4.2 In-bore tri-modal 3D imaging

In order to assess the in-bore tri-modal 3D imaging capability of the developed system, *in vivo* 3D PA/US was performed inside the MRI bore with the MRI scanning sequence running. The living mouse was installed as depicted in Fig. 7. Its tail was targeted using 2D PA/US, and the whole body of the mouse was scanned with MRI as a global image. The MRI images corresponding to the PA/US slices were identified by referring to the installed MRI markers and the geometrical structure of the PA/US probe.

**Fig. 13:**
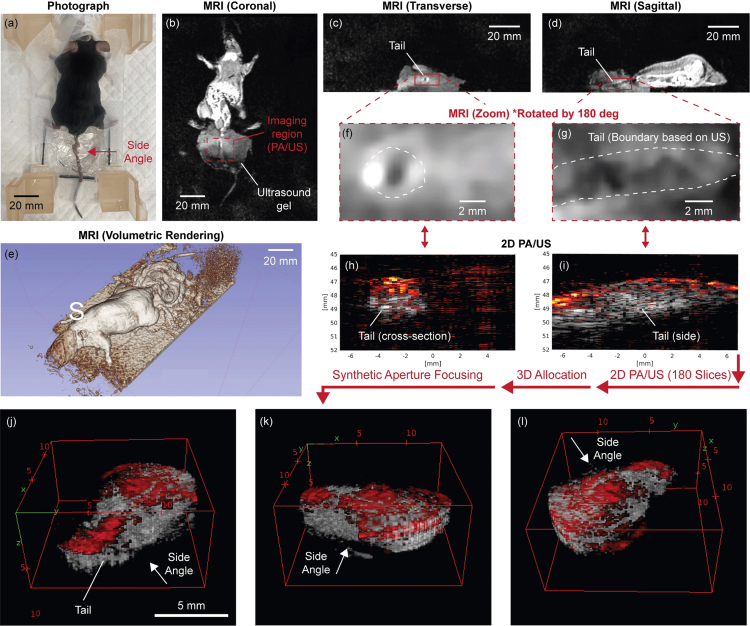
Results of *in vivo* in-bore tri-modal 3D imaging; (a) Photograph of the prepared living mouse, (b)–(d) Global MRI images for coronal, transverse, and sagittal views, (e) Volumetric-rendered MRI, (f)–(g) Magnified and rotated MRI images corresponding to 2D PA/US images shown in (h) and (i) (Dynamic range for 2D US is 30 dB) ((f) and (g) were identified based on the anatomical feature, installed MRI markers, and the geometrical feature of the PA/US probe in the images. The positions and the scales of the MRI images were roughly aligned and not exactly the same as the corresponding PA/US images.), (j)–(l) 3D PA/US images in three different views.

The MRI images (Fig. 13(b)–(d)) demonstrate successful capture of the complete mouse anatomy across all angles. While the ultrasound gel is visible in the MRI images, the tail remains delineated within the gel medium. As particularly evident in Fig. 13(d), although artifacts are generated from the PA/US probe, their impact on the imaging area is sufficiently small compared to the imaging stage. Fig. 13(f) and (g) represent magnified and rotated MRI images to correspond with the 2D PA/US images shown in Fig. 13(h) and (i). While the current MRI imaging sequence makes tail identification challenging at this magnification scale, the corresponding US imaging clearly delineates the tail structure, with additional signal confirmation in the PA imaging. These 2D images were acquired through probe rotation, collecting over 180 images to generate the 3D visualization shown in Fig. 13(e).

These results demonstrate that our developed device successfully achieves simultaneous MRI imaging, rotational actuation for 3D imaging, and PA/US imaging even in an *in vivo* environment.

## 4 Discussion

This research investigated the MRI-compatible transrectal US and PA imaging system, including its actuation module, assuming the application to PCa. The performance of this system has been evaluated within both the phantom and the *in vivo* settings.

The engineered PA/US probe utilizes a reflector-based mechanism to facilitate transrectal PA/US, an approach corroborated in the US imaging by Tsumura et al. [36]. Their research verified that the reflector’s presence does not substantially degrade image quality, as evidenced by a comparative analysis of images taken with and without the reflector. In the current study, the PA functionality was introduced through the integration of an optical fiber. Given that the incorporated dielectric mirror consistently maintains an average reflection rate exceeding 96% within the wavelength range used in this experiment, it suggests that the PA imaging quality is not significantly compromised by the reflector.

During both the phantom experiment and the *in vivo* experiments, the imaging targets were positioned as such to avoid artifact interference in the MRI image from the developed hardware, leading to its successful visualization in the MRI image. The majority of the artifact appears to be associated with the brass material around the transducer. The replacement of the brass material with a non-metallic substrate, such as ULTEM used in the outer shaft, is anticipated to curtail the artifact. As the underlying concept will be implemented in real-world clinical contexts, minimizing such artifacts and securing an expansive FOV becomes essential.

One of the core aims of the present study is to facilitate the accompanying target visualization of PA, US, and MRI, with no requirement for patient movement between scans. Besides the necessary MRI compatibility of the PA/US probe, a fundamental prerequisite for the system is its capacity for image registration of all three modalities. Several potential methodologies exist for image registration, one of which involves identifying the PA/US imaging slice within the MRI image, informed by the geometric characteristics of the probe and MRI markers visualized via MRI. The addition of reference points, such as an MRI marker as used in the *in vivo* experiments, may aid in discerning the geometric relationship between the probe and the MRI image. Another approach would be to consider the similarity of the image of each modality. To make Fig. 11(b), the first approach was used, and the MRI image displayed was manually scaled and aligned by referencing the corresponding US image (Fig. 11(c)). For a smoother workflow, this process should be automated in future work as demonstrated in some existing MRI-US FUSION platforms [37].

While the proposed system exhibited anticipated performance characteristics, several limitations should be considered. First, water or transparent ultrasound gel was utilized as the acoustic coupling medium throughout the experiments. This circumstance offers a more advantageous scenario than that provided by actual biological tissues in terms of depth penetration, especially for PA. Towards the translation into real-world clinical settings, improving the PA’s depth penetration is crucial to secure a wider FOV. Potential countermeasures include adding an optical fiber independently from the transducer, passing it through the urethra for instance, and directing a laser proximal to the target, or increasing the core diameter of the optical fiber to facilitate the delivery of enhanced laser energy. Second, although the *in vivo* study was conducted, the animal model can be improved. For more clinically relevant validation, future studies should employ PCa and PSMA-targeted contrast agents. One of the examples of such PSMA-targeted contrast agents is proposed and its performance is demonstrated by Lesniak et al. [38]. Since the proposed contrast agent relies on Cy7.5 for its contrast, our *in vivo* validation using Cy7.5 suggests the high potential efficacy of such a PSMA-targeted contrast agent with our developed device. The implementation of these more appropriate models would enhance the clinical translatability of our platform.

## 5 Conclusions

In this research, we designed and developed an MRI-compatible PA/US imaging platform that enables in-bore tri-modal imaging for PCa applications. The results demonstrate the system’s ability to operate safely within the MRI environment while maintaining imaging performance across all three modalities. The phantom and *in vivo* studies confirmed the feasibility of in-bore tri-modal imaging, including spectroscopic PA capabilities and 3D PA/US imaging. While several technical aspects require refinement in future work, such as PA imaging depth and automated registration, these results validate the in-bore tri-modal imaging concept from multiple perspectives, providing substantial support for its feasibility in clinical translation.

## CRediT authorship contribution statement

**Ryo Murakami:** Writing – review & editing, Writing – original draft, Visualization, Validation, Software, Project administration, Methodology, Investigation, Formal analysis, Data curation. **Yang Wang:** Writing – review & editing, Validation, Investigation. **Wojciech G. Lesniak:** Writing – review & editing, Resources, Conceptualization. **Ryosuke Tsumura:** Writing – review & editing, Software, Resources, Methodology, Conceptualization. **Yichuan Tang:** Writing – review & editing, Visualization, Validation, Software, Investigation. **Shang Gao:** Writing – review & editing, Visualization, Software. **Yasuyuki Tsunoi:** Writing – review & editing, Resources, Methodology. **Christopher J. Nycz:** Writing – review & editing, Methodology, Conceptualization. **Martin G. Pomper:** Writing – review & editing, Funding acquisition, Conceptualization. **Gregory S. Fischer:** Writing – review & editing, Supervision, Resources, Methodology, Conceptualization. **Haichong K. Zhang:** Writing – review & editing, Supervision, Software, Resources, Project administration, Methodology, Investigation, Funding acquisition, Conceptualization.

## Declaration of Generative AI and AI-assisted technologies in the writing process

During the preparation of this work, the authors used ChatGPT, Claude, and GitHub Copilot. After using this tool, the authors reviewed and edited the content and take full responsibility for the content of the publication.

## Declaration of competing interest

The authors declare the following financial interests/personal relationships which may be considered as potential competing interests: Haichong K. Zhang reports financial support was provided by National Institute of Health. Ryo Murakami reports travel was provided by IEEE. Martin G. Pomper reports financial support was provided by National Institutes of Health. Haichong K. Zhang, Gregory S. Fischer, Ryosuke Tsumura, Yang Wang, Ryo Murakami has patent pending to NA. If there are other authors, they declare that they have no known competing financial interests or personal relationships that could have appeared to influence the work reported in this paper.

## Figures and Tables

**Fig. 14:**
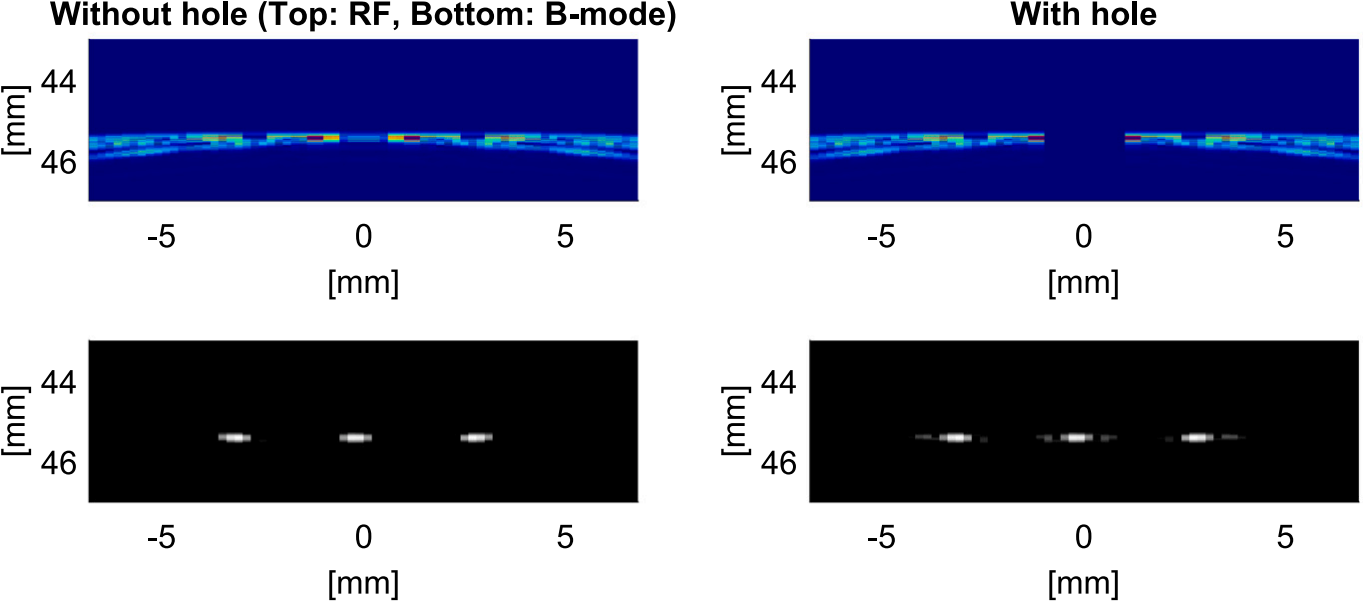
Radiofrequency data and B-mode images of Field II simulation analysis regarding the hole influence on US imaging.

**Fig. 15:**
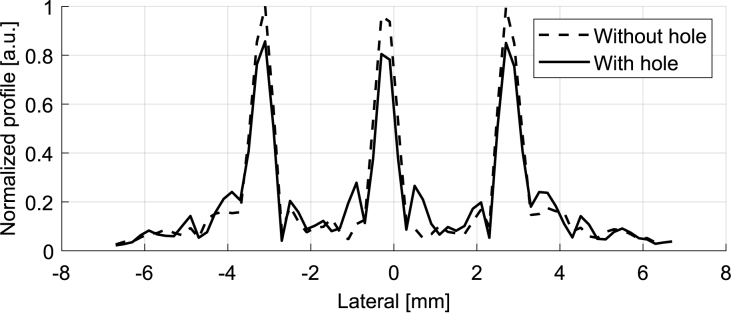
Beam profile of the B-mode images from Field II simulation analysis.

**Fig. 16:**
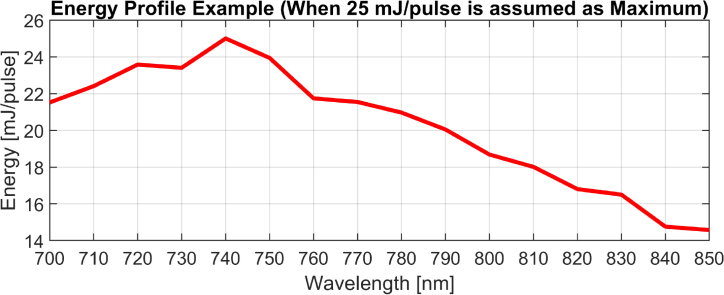
Fiber laser energy output example (When 25 mJ/pulse is assumed as maximum).

## Data Availability

The data that has been used is confidential.

## Appendix A Hole impact analysis

A simulation analysis is performed to estimate how the existence of the hole for the laser affects the resulting US/PA image. The Field II simulation software [39], [40] is used and the parameters used for the simulation are summarized in Table 1. The existence of the hole is simulated by eliminating the signals received by the corresponding transducer elements, which can be considered to be the most disadvantageous condition. In reality, although there exists an approximate 12 dB disparity between the maximum and minimum gain values among the elements, an examination of the manufacturer-provided specifications indicates that a certain degree of signal transmission and reception is feasible, even in areas where the hole reduces the element surface area. In the results simulating the US imaging with three scatters at the depth of 45 mm (Fig. 14, Fig. 15), as an example case, the existence of the hole does not affect the lateral resolution. On the other hand, the signal intensities at the lateral center and ±3mm away from the center get weaker by approximately 20% and 15%, respectively. In addition, it is observed that the relative amount of the side lobes around the center signal is enlarged. Although the hole’s influence can vary as a function of the position of a signal source in the three-dimensional space, the results obtained in this section are treated as the representative result and the influence of the hole is basically ignored in this study unless there is unacceptable influence caused by the hole.

## Appendix B Laser details

The details of the laser characteristics are summarized in this section to secure the reproducibility and discuss the safety of the developed device. This safety assessment was performed by referring to the American National Standard for Safe Use of Lasers (ANSI Z136.1), published by the American National Standards Institute (ANSI) [41].

The observed maximum laser energy in the wavelength range used in this study is estimated to be less than 25 mJ/pulse. The pulse repetition rate in this study was fixed at 20 Hz with a pulse width of 5 ns. Fig. 16 represents the example of the laser energy emitted from the optical fiber. 25 mJ/pulse is assumed as the maximum, and the entire profile is scaled with the maximum. Unless otherwise specified, PA frames per slice and wavelength were averaged to improve the SNR. Please note that the number of frames contributing to each averaged PA image may deviate from the intended target numbers due to laser output instability or limitations in the data processing pipeline. Based on the camera-based visual inspection under the conditions simulating the *in vivo* experiments, the optical spot size at the tissue interface was estimated to be approximately 25 mm in diameter. Based on these conditions, the single-pulse fluence is computed as 5.1 mJ/cm${}^{2}$, which is below the effective maximum permissive exposure (MPE) for this condition, 20 mJ/cm${}^{2}$ (at 700 nm). Since the 700 nm wavelength poses the lowest MPE in this wavelength range, the experimental parameters for this aspect were confirmed to be in compliance with the safety standard.
